# Neurology APP Residency Training Programs: We Are on Our Way!

**DOI:** 10.1212/NE9.0000000000200039

**Published:** 2023-03-07

**Authors:** Joel Morgenlander

**Affiliations:** From the Department of Neurology, Duke University School of Medicine, Durham, NC.

The current need for improved access to health care spans all specialties including neurology. To improve access to care, our healthcare teams are increasing their complement of advanced practice providers (APPs), including nurse practitioners (NPs) and physician assistants (PAs).^1,2^ The focus of APP primary training programs is on general medical education with particular focus on various concentrations for NPs. Given our need to use APPs to expand our clinical access in neurology, it is reasonable to ask what neurologic exposure APPs gain during their primary training programs.

In this issue of *Neurology*® *Education*, Harrison et al.^3^ examine the extent of neurologic education presented to trainees in US PA programs. This was performed by a combination of surveying the individual program directors and reviewing online website data about the individual programs. Attempts were made to understand preclinical neuroscience course work, instruction in neurologic examination techniques, and clinical opportunities available through neurology rotations.

While the response rate of the program directors was low at 23.5%, important information was gathered from these sources that paints the picture of insufficient neurologic education to prepare these PAs to confidently perform well directly out of training in a neurologic clinical environment. Of the websites reviewed, only 20.8% had neuroscience didactics listed. Neurologic physical diagnosis was rarely taught by a neuroscience clinician. While 85% of program directors indicated their program had an elective in neurology available, it appeared a minority of students took advantage of that option. The authors demonstrated that more time was spent on cardiology than neurology commensurate with the idea that the educational focus is on training the students for the practice of primary care. It was appropriately believed that additional neurologic education during PA programs would be beneficial to the neurologic workforce.

It is very important that PA programs include neurologic education in their curriculum. Many of the PAs not practicing in a primary neurologic setting will be confronted by neurologic issues in their patients. By leveraging neuroscience provider teaching of neurologic examination and disorders, they may be able to improve the quality of education for their students. Those interested in neuroscience practice do benefit from elective experiences.

From my perspective, the biggest bang for the buck is to create Neurology APP Residency Training Programs.^4^ Let us look at the parallel with training neurologists and primary care physician training. The American Board of Internal Medicine board examination blueprint states that 4% of the test is about neurology.^5^ Similarly, the American Board of Family Medicine board examination blueprint states that 3% of that test is about neurology.^6^ Neither board requires a neurology rotation. For comparison, the Physician Assistant National Certifying Examination blueprint includes 7% of the test allocated to neurology.^7^ Neurology residencies were created to prepare physicians to learn the breadth of neuroscience information needed to provide outstanding neurologic clinical care.

Across the United States, Neurology APP Residency Training Programs have started and are increasing in numbers. The program directors have begun to meet to develop core curricula and share resources to enhance the opportunities for both PA and NP graduates to gain further education in neurology along their path to be providing neurologic services as part of an expanded team. Ultimately, the program directors will search for an appropriate accreditation partner for APP residency programs and trainees. These programs are efforts in addition to the substantial work by the American Academy of Neurology^8^ and others to increase neurologic educational opportunities for APPs. Similar to the evolution of neurology residency programs from internal medicine, neurology residency training for APPs will better serve our neurology APPs and our patients.

Editor's Note: This was previously posted online as an Editorial Blog on December 21, 2022.
